# Veratrum Viride, or American Hellebore

**Published:** 1853-02

**Authors:** 


*Veratrum Viride, or American Hellebore.* By W. C. Norwood, M. D., of Cokesbury, South Carolina.

The past numbers of this Journal have contained several articles by Dr. Norwood, on the sedative influence of the veratrum viride, and its successful adaptation to the treatment of a variety of affections accompanied by febrile movement, or marked excitement of the circulation. In the January number of the Southern Med. and Surg. Journal, we find a long article on the ubject, giving reports of numerous cases in which its remedial action appeared to be signally successful. We select a portion of this article, containing an account of the properties of the drug, the mode of preparation for medicinal use, etc. The testimony made to the value of the remedy is amply sufficient to commend it to the attention of the profession.—EDITOR BUFFALO MEDICAL JOURNAL.

Veratrum viride, green hellebore, American hellebore, is not our common poke-root or *Phytolacca Decandra*, but is the poke-weed, veratrum viride, and is entirely different in its appearance and properties. Again—it is called white hellebore, by the Shakers, and those ordering the veratrum viride often get the white hellebore proper, or European, for it, by not being specific in the correction of the error in name. The properties and powers of veratrum viride are the following:

1st, acrid—This property is very limited and confined to the fauces.

2d. It is adanagic, deobstruent or alterative: this property it possesses in a marked and very high degree; not equalled by calomel or iodine in this particular, which will adapt it to the relief and cure of many diseases hitherto beyond the reach of any remedy. Of this class of diseases, those which we think will be much benefitted by it, are, cancer and consumption.

3d. It is actively and decidedly expectorant, so much so that we rarely add any other article.

4th. It is one of the most certain diaphoretics belonging to the materia medica: it often excites great coolness or coldness of the surface; in some cases the skin is rendered merely soft and moist; in other instances, the perspiration is free, and at other times it is most abundant; but, notwithstanding its profuseness, it does not reduce or exhaust the system, as many diaphoretics do when in excess, and therefore need not excite alarm nor be suspended on that account.

5th. It is nervine, not narcotic, under any circumstances; as since our first article, we have taken it more than twenty times to test its varied powers, and we have taken it in all quantities, from the production of free emesis down to the minimum dose. This property renders it of great value in the treatment of painful diseases and such as are accompanied with convulsions, morbid irritability and irritative mobility. For example—pneumonia, rheumatism, puerperal fever, convulsions generally, and palpitation of the heart, &c.

6th. It is one of the most certain and efficient emetics known, and is peculiarly adapted to meet that indication in hooping cough, asthma, croup, scarlet fever, and in all cases where there is much febrile and inflammatory action. It often excites severe nausea and frequent vomiting, which, taken in connection with great paleness, often alarms the patient and by-standers; but these effects, when in excess, are readily relieved by one or two full portions of morphine and tinct. of ginger, or of laudanum and brandy. One grand and leading feature is, that the exhaustion which follows it, is not excessive and permanent, but confined merely to the effort. Again, the matter first ejected, is a large quantity of thick, slimy mucus, and soon after, the liver is called on to pour forth its own fluid in abundance.

7th. The seventh property is its most valuable and interesting, and for which it stands unparalleled and unequaled as a therapeutic agent. So much has already been written on what we call the sedative—arterial sedative—properties of the agent, or the power it possesses of controlling and regulating arterial action, that we shall not again run over the amount of evidence on this part of the subject. By virtue of this and other powers, the treatment of disease has been much simplified, and when the effects recorded in the case of Mr. G.'s negro woman, shall have been fully considered, we may bid adieu to much of the supposed necessity for stimulants in the treatment of atonic or asthenic cases. We challenge the medical world to produce its equal, as a therapeutic agent, for certainty of effect, for extent of effect, or for peculiarity of effect, and the ease and safety with which it may be administered to small and great. In small portions, we have found nothing to equal it in exciting and promoting appetite.

The formula we use is the following:

℞ Root of veratrum viride, dried, ℥viii.
Alcohol, of the shops, undiluted, ℥xvi.

Let it stand from ten days to two weeks. Medium dose for an adult male, eight drops, to be increased one or two drops every portion, until nausea or vomiting, or a reduction in the frequency of the pulse takes place: then reduce one-half in all cases. Females, and persons from fourteen to eighteen years of age, should commence with six drops, and increase as above. Children, from one to two years of age, to commence with one drop; from two to five years of age, two drops, and increase one drop. The usual interval with us is three hours between the portions. In ordinary cases of pneumonia, we usually continue it three days after the symptoms are subsided. In typhoid fever, and many other diseases, it requires to be continued much longer. For the satisfaction and information of the profession, we would state that it may be continued indefinately, or any length of time, in moderate doses, or short of nausea, without the least inconvenience. The only objection that could be urged, is the increase of appetite, or desire for food. It is not cathartic—it is like all other remedial agents, subject to the same rules and regulations, making it out of the question for a person to lay down any but general directions for regulating the dose. We are better pleased with the method adopted for getting its first impression by Dr. Welburn, of Farmville, Alabama, than with our own. We allude to the short interval between the first three portions he administers: He gives "six drops; in ten minutes, seven drops; in ten minutes more, eight or ten drops; and then suspends the dose till vomiting occurs," which will be sure to take place in a large majority of cases. In the outset of many cases, we would recommend Dr. Welburn's manner of using it. In a male, twenty-five drops is the largest quantity we have known to be required to excite emesis, and sixteen drops in the female when given in the manner and at the intervals we have directed. There need be no danger apprehended of its exciting inflammation of the stomach —we have given special attention to that particular. It is peculiar and at the same time interesting in its effects. The fact of its acting as a sedative on almost every other portion of the system, diminishing the vascular and muscular action and motion of every other part, and increasing that of the stomach. We have seen it produce emesis in very susceptible persons, and the contractions of the stomach were so rapid as to be almost continuous and uninterrupted; but a strong alcoholic tincture of ginger and morphine would afford more prompt and immediate relief than any other article that we have ever used. We have never seen a case that failed to be relieved by the above remedies in thirty minutes. The great advantage of the remedy is that it does not exhaust longer than the effort to vomit is concerned. A great many remedies leave the patient in an exhausted and enfeebled condition, aside from the effort or immediate action—not so with the veratrum viride. Again, tartar emetic should never be given with it, in any form or manner. The only cases in which we have seen the tincture of veratrum viride purge, were when given in combination with tartar emetic, or with Coxe's hive syrup. In most of these cases it excited a violent cholera-morbus. We would not think of giving the tincture of veratrum viride where tartar emetic had been used, without preceding it with a full dose of morphine or laudanum at least one hour. We have known many fall out with the veratrum viride when it was not at fault. Again, venesection, when a large quantity of blood is drawn, increases materially its effects, whereas opium and morphine lessens or diminishes them. If a patient had been bled freely, preceded or followed by a liberal use of tartar emetic, and then followed up with medium portions of the tincture of veratrum viride, we should anticipate and prepare for drastic, if not hazardous effects.

